# Cryptic mtDNA Diversity of *Diopatra cuprea* (Onuphidae, Annelida) in the Northwestern Atlantic Ocean

**DOI:** 10.3390/biology12040521

**Published:** 2023-03-30

**Authors:** Erik E. Sotka, Tina Bell, Sarah Berke

**Affiliations:** 1 Department of Biology, College of Charleston, Charleston, SC 29412, USA; 2 Department of Biology, George Mason University, Fairfax, VA 22030, USA; 3 Department of Biology, Siena College, Loudonville, NY 12309, USA

**Keywords:** Annelida, Onuphidae, ecosystem engineer, phylogeography

## Abstract

**Simple Summary:**

Molecular tools continue to reveal cryptic biodiversity within common and ecologically important species. The decorator worm *Diopatra cuprea* is an ecosystem engineer of intertidal beds of high-salinity estuaries of the Atlantic and Gulf of Mexico shorelines. Here, we sequenced mitochondrial cytochrome oxidase I (COI) in *D. cuprea* populations and discover evidence for several deep mitochondrial lineages, suggesting the presence of cryptic diversity.

**Abstract:**

Marine annelid taxonomy is experiencing a period of rapid revision, with many previously “cosmopolitan” species being split into species with more limited geographic ranges. This is exemplified by the *Diopatra* genus, which has recently witnessed dozens of new species descriptions rooted in genetic analyses. In the northwestern Atlantic, the name *D. cuprea* (Bosc 1802) has been applied to populations from Cape Cod through the Gulf of Mexico, Central America, and Brazil. Here, we sequenced mitochondrial cytochrome oxidase I (COI) in *D. cuprea* populations from the Gulf of Mexico to Massachusetts. We find evidence for several deep mitochondrial lineages, suggesting that cryptic diversity is present in the *D. cuprea* complex from this coastline.

## 1. Introduction

Annelid taxonomy is experiencing a period of rapid revision at every level, from individual species to the entire phylum [1,2,3,4]. Historically, taxonomists of the 19th and early 20th centuries embraced the ‘cosmopolitan species concept’, believing that marine annelid faunas were dominated by a relatively small number of very widespread species. This view began to crumble in the face of increasingly detailed morphological work beginning in the 1970s, and has now fully collapsed under evidence from modern phylogenetic methods (reviewed by [3]). While we now understand that annelid diversity is vastly greater than was once thought, the work of fully understanding that diversity remains very much a work in progress.

The problem of refining “cosmopolitan” taxa is especially well-illustrated in the polychaete genus *Diopatra.* Historically, the two best-known species—*D. neapolitana* from western Europe and *D. cuprea* from eastern North America—were applied to far-flung onuphids from Africa to India, Southeast Asia, and Australia [5,6]. Systematists have been working at this problem since at least the 1980s [7,8], with over a dozen new *Diopatra* species described in the past decade alone, and more to come [9,10,11,12,13,14,15,16,17,18,19,20]. However, one of the ecologically best-known species, *D. cuprea* (Bosc 1802), has not yet been evaluated using modern phylogenetic methods.

*D. cuprea* occurs intertidally on the US east coast from Duxbury MA southward (the southern limit is not well established) [21,22]). *D. cuprea* provides one of the best-described examples of ecosystem engineering in estuarine sediments; the worm builds robust tubes that descend 1 m or more vertically into the sediment, with an above-sediment tube-cap emerging 2–5 cm above the sediment surface. This tube-cap is characteristically decorated with fragments of drift algae, shell, and other debris. The tube stabilizes sediment, alters water flow across the sediment surface, and physically excludes epibenthic predators. Together, these physical effects increase the diversity and abundance of nearby infauna, and of the epifauna living on the tube itself (reviewed by [22]). By attaching to algae, the worm creates an algal canopy in habitats where attached algae would otherwise be rare [23], and this behavior is facilitating the invasive red alga *Gracilaria vermiculophylla* throughout most of the US east coast [24,25,26,27]. *D. cuprea’s* emergent, decorated tube is typical of the genus, and our model for understanding the genus’ ecological role worldwide is largely informed by studies of *D. cuprea* [22].

The *D. cuprea’s* type locality is in South Carolina, but the name has been applied to *Diopatra* from Cape Cod through Brazil [10,20]. *Diopatra* from Brazil have recently been redescribed with four new species, none of which were *D. cuprea* [20], consistent with a non-cosmopolitan range for this species. The possible existence of cryptic *Diopatra* diversity in the northwestern Atlantic remains currently unexplored. *D. cuprea* is known to exhibit a curious latitudinal gradient in its tube-decorating behavior, with worms in Florida decorating far less than worms between Cape Cod and Georgia, even when offered the most commonly utilized algae in controlled conditions [21]. This biogeographic pattern in behavior raises the hypothesis that there may be hidden population-level genetic diversity in this region. Here, we sequenced a portion of the mitochondrial cytochrome oxidase I (COI) gene from *D. cuprea* populations from the Gulf of Mexico to Massachusetts in order to assess population genetic structure and any cryptic diversity.

## 2. Materials and Methods

Per site, 10–40 samples were collected by hand, placed into 95% ethanol and returned to the College of Charleston Marine Laboratory in Charleston SC in approximately 2009–2012 (Table 1). Samples were identified using morphological traits previously described [6,28], and extracted using a Nucleospin Tissue and Blood Kit (Maschery-Nagel), with the manufacturer’s recommended protocol. A portion of the COI gene was then PCR amplified using the protocol in [29]. These were cleaned with an EXO-SAP-IT protocol and sent for Sanger sequencing with these same primers at a private company.

The sequences were trimmed and quality-checked with *4Peaks* software v1.8 (nucleobytes.com) and saved as a fasta-formatted file. This yielded a dataset of 539 nucleotides across 153 individuals (GenBank Accession Numbers OQ700009—OQ700161). We used *ape 5.0* [30] and a custom code in R v4.2.3 [31] to estimate the genetic distance between individuals, and assigned labels to all 15 unique haplotypes (Hap 1–15). To assess the placement of northwestern Atlantic *D. cuprea* into a wider phylogeographic context, we used *MUSCLE* v3.8.31 [32] with default parameters embedded in SeaView 5.0.5 [33] to align our dataset and sequences from published sources (Table 2). We ran *PhyML* 20120412 [34] as implemented in SeaView to estimate the phylogenetic relationships with default parameters (GTR + 4 rate classes as a model of evolution; 100 bootstrap replicates). We also generated a Bayesian analysis with *MrBayes* 3.2.7a [35] with 10,000 generations, a sampling frequency of 100, and a burn-in period of 250 generations. We used *R::ape* to visualize the Bayesian tree. We also used *strataG* 1.0 [36] to assess nucleotide diversity, pairwise Weir and Cockerham’s F_ST_ and the proportion of unique haplotypes within populations. Finally, we used linear regressions to assess the relationship between latitude and nucleotide diversity, and the latitude and proportion of unique haplotypes.

## 3. Results

The within-population nucleotide diversity ranged from <0.001 to 0.101 and the proportion of unique haplotypes per populations ranged from 7 to 67% (Table 1). There was no linear relationship of nucleotide diversity with latitude (r^2^ = 0.317; F_1,9_ = 4.185; *p* = 0.071) nor proportion of unique haplotypes with latitude (r^2^ = 0.015; F_1,9_ = 0.1367; *p* = 0.720).

**Figure 1:**
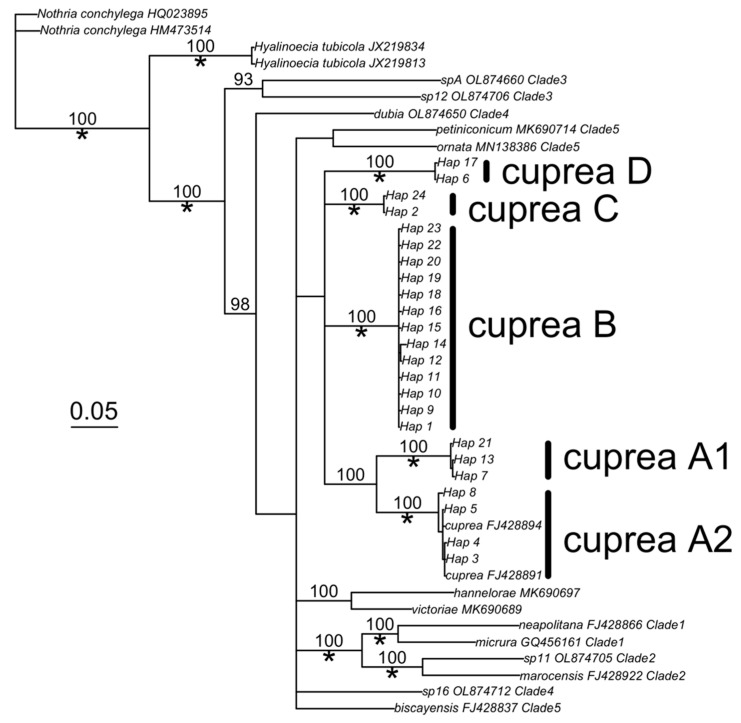
Cryptic *Diopatra cuprea* genetic diversity. The Bayesian phylogeny was based on 550 bp of cytochrome *c* oxidase I. Numbers on edges indicate posterior probability, while asterisks indicate 90% or greater maximum likelihood bootstrap support (100 replicates). Clade designations (i.e., numbers after taxa) were defined using the work of [12]. An approximate 5% divergence between haplotypes is indicated by the segment.

All the COI haplotypes sequenced from morphologically similar *Diopatra cuprea* from the east coast of the United States (Gulf of Mexico to Massachussetts) clustered into a single monophyletic clade, although the clade had minimal statistical support (i.e., ML bootstrap and Bayesian posterior probabilities were less than 90%). None of these samples clustered within Clades 1–3 from the work of Hektoen et al., 2022 [12]. Instead, samples were within a lineage labeled as Clade 4–5 of Hektoen et al., 2022 ([12]; Figure 1).

**Figure 2:**
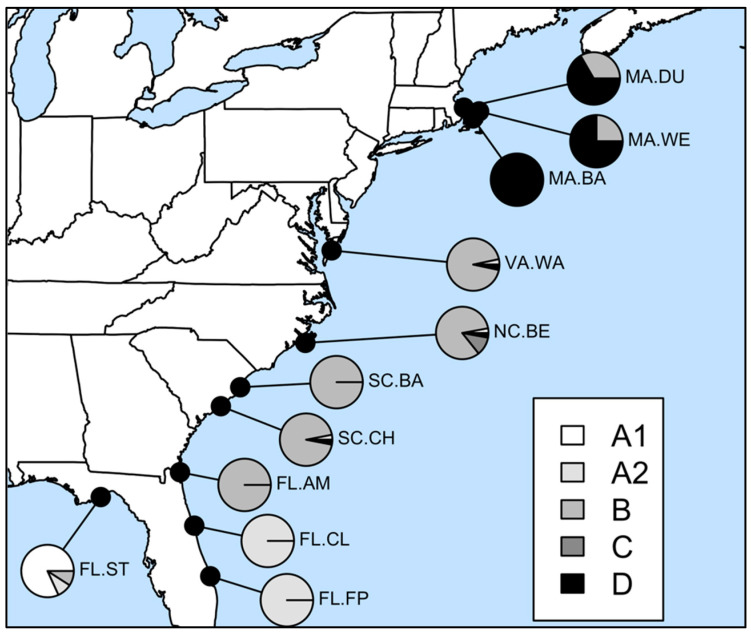
Geographic distribution of COI clades (labeled A1-D; see Figure 1) of *Diopatra cuprea*.

Within the northwestern Atlantic *D. cuprea* samples, we detected five divergent lineages that differed between 14.6 and 20.5% (Table 3). We labeled these *D. cuprea* clades based on their relationship to each other and their geographic distribution (Figure 2; see clade frequencies in Table 1). A1 and A2 are a monophyletic group found mainly in Florida; A1 dominated the St. Thersa Beach population in the Gulf of Mexico (81% of haplotypes), while A2 dominated the southeastern coasts of Florida (100% of haplotypes from Fort Pierce and Chicken Island). Clade B dominates the mid-Atlantic states, from northeastern Florida (Amelia Island) to Virginia, and rarely occurred in other locations. Clade C was only found in North Carolina and Clade D dominated Massachusetts populations, and rarely occurred in mid-Atlantic states.

The overall F_ST_ across all populations was 0.728 (*p* < 0.001) and the overall F_ST_ across the four regions (FL-Gulf; FL-Atlantic; mid-Atlantic; MA) was 0.826 (*p* < 0.001). Table 4 and Table 5 indicate the pairwise F_ST_ among the populations and regions, respectively.

## 4. Discussion

In morphological terms, all the individuals collected from the Gulf of Mexico through Massachusetts are consistent with the currently described *Diopatra cuprea* (Bosc 1802). While we found no statistical evidence for a monophyletic clade of *D. cuprea* in the northwestern Atlantic, all five COI clades were clustered away from the recently defined Clades 1, 2 and 3 in the work of Hektoen et al., 2022 [12]. This paper utilized two mitochondrial (COI and 16s rDNA) and one nuclear locus (28S rDNA) to assess phylogenetic relationships; it is likely that the application of these two other loci would allow greater resolution of the *D. cuprea* lineages and their exact placement within the wider phylogeny presented in the work of Hektoen et al., 2022 ([12]; see [11]). We also note that the type locality of *D. cuprea* was South Carolina, which is strongly dominated by Clade B [19].

The phylogeography of the widespread *Diopatra cuprea* mirrors that of several nearshore and estuarine species in North America. The monophyletic split between the A1 and A2 versus the others (B, C and D) likely reflects the Gulf of Mexico vs. eastern seaboard, which now have a secondary contact zone in eastern Florida near Cape Canaveral [40,41,42], and was driven by the separation of these water bodies during the Pliocene or Pleistocene. The split between Massachusetts Clade D from the mid-Atlantic clades (B and C) likely mirrors historical separation during the Pleistocene [43] and has a secondary contact zone near the New Jersey/New York border [44]. There are few species whose geographic range spans the Gulf of Mexico through New England; of these, one that displays similar phylogenetic breaks is the monocot *Spartina alterniflora* [45], which lives in close proximity to the high-salinity marshes that also constitute *D. cuprea* habitats.

The populations were not reciprocally monophyletic. Each population was dominated by one or two COI clades, but most populations harbored 1–2 other clades at low frequency. As examples, the Gulf of Mexico population has a low frequency of the mid-Atlantic B clade, while a North Carolina population has a low frequency of the Gulf of Mexico A1 clade and Massachusetts D clade. These may reflect low-frequency dispersal events between the regions, incomplete lineage sorting, or a mix of both.

Low-frequency dispersal events could occur naturally or via human-assisted transport. Unlike *Diopatra* in Europe, *D. cuprea* is not harvested for bait in the US (most likely because it is more difficult to obtain than *D. neapolitana* [22]). Transport as a bait worm is, therefore, unlikely. *Diopatra*’s larval period is quite short, making transport in ballast unlikely [46]. If human-assisted transport plays any role in these distributions, it is most likely via the transport of newly settled juveniles in mud associated with bivalve aquaculture. This is also the mechanism proposed for the *D. biscayensis* disjunct distribution in France [46]. Galaska et al. [46] found that individual *D. biscayensis* in the disjunct population were disproportionately found near the ropes used for mussel culture. None of our sampling sites were (to our knowledge) especially close to commercial aquaculture sites, but bivalve aquaculture certainly does occur throughout the eastern United States. We can tease apart the relative importance of lineage sorting and more recent dispersal by assessing the phylogeographic patterns in the nuclear genome (e.g., [38]). Moreover, future sampling efforts could also evaluate whether rare clades are associated with aquaculture sites.

Overall, our results provide preliminary evidence that cryptic diversity is indeed present in *D. cuprea* populations of the northwestern Atlantic. This is consistent with the explorations of other *Diopatra* populations in other parts of the world.

## Figures and Tables

**Table 1:** Populations collected. We indicate the regions (see text), latitude and longitude, alternative IDs, collector (Sarah Berke or Tina Bell), sample size (*n*), COI haplotype clade frequencies, nucleotide diversity and proportion of unique haplotypes.

ID	Population	Region	Latitude	Longitude	Collector	Total (*n*)	A1 (*n*)	A2 (*n*)	B (*n*)	C (*n*)	D (*n*)	Nucleotide Diversity (mean)	Unique Haplotypes (%)
FL.ST	Saint Teresa Beach FL	FL-Gulf	29.922381	−84.474212	Bell	11	9	1	1	0	0	0.052	0.545
FL.FP	Fort Pierce FL	FL-Atlantic	27.4575	−80.306	Berke	7	0	7	0	0	0	0.000	0.143
FL.CL	Chicken Island FL	FL-Atlantic	29.03239	−80.91479	Berke	4	0	4	0	0	0	0.000	0.250
FL.AM	Amelia Island Florida	FL-SC-NC-VA	30.69617	−81.46038	Berke	5	0	0	5	0	0	0.002	0.600
SC.CH	Charleston SC	FL-SC-NC-VA	32.75106	−79.90281	Berke	33	0	1	31	0	1	0.018	0.303
SC.BA	North Inlet SC	FL-SC-NC-VA	33.341	−79.165	Berke	7	0	0	7	0	0	0.000	0.143
NC.BE	Beaufort NC	FL-SC-NC-VA	34.72195	−76.687	Berke, Bell	35	1	0	29	4	1	0.044	0.171
VA.WA	Wachapreague VA	FL-SC-NC-VA	37.601553	−75.686788	Berke	30	0	1	28	0	1	0.020	0.167
MA.BA	Barnstable Harbor MA	MA	41.70911	−70.30297	Berke	14	0	0	0	0	14	0.000	0.071
MA.WE	Wellfleet MA	MA	41.929924	−70.073089	Bell	4	0	0	1	0	3	0.076	0.500
MA.DU	Duxbury MA	MA	42.04647	−70.65025	Berke	3	0	0	1	0	2	0.101	0.667

**Table 2:** GenBank accession numbers of COI for *Diopatra* spp. and other polycheates.

Species	CladeID-Hektoen	Accession Num	Reference
*Diopatra_biscayensis*	5	FJ428837	[29]
*Diopatra_ornata*	5	MN138386	[37]
*Diopatra_petiniconicum*	5	MK690714	[31]
*Diopatra_cuprea*	5	FJ428891	[29]
*Diopatra_cuprea*	5	FJ428894	[29]
*Diopatra_victoriae*	5	MK690689	[31]
*Diopatra_hannelorae*	5	MK690697	[31]
*Diopatra_marocensis*	2	FJ428922	[29]
*Diopatra* sp11	2	OL874705	[38]
*Diopatra_micrura*	1	GQ456161	[16]
*Diopatra_neapolitana*	1	FJ428866	[29]
*Diopatra* sp12	3	OL874706	[6]
*Diopatra* spA	3	OL874660	[6]
*Diopatra_dubia*	4	OL874650	[6]
*Diopatra* sp16	4	OL874712	[6]
*Nothria_conchylega*	Outgroup	HM473514	[10]
*Nothria_conchylega*	Outgroup	HQ023895	[10]
*Hyalinoecia_tubicola*	Outgroup	JX219813	[39]
*Hyalinoecia_tubicola*	Outgroup	JX219834	[39]

**Table 3:** Mean percent difference between COI haplotype clades.

	A1	A2	B	C	D
A1	-				
A2	0.146	-			
B	0.178	0.169	-		
C	0.167	0.169	0.151	-	
D	0.183	0.205	0.172	0.192	-

**Table 4:** Pairwise PhiST (below diagonal) and *p*-values (above diagonal) among populations.

	FL.ST	FL.FP	FL.CL	FL.AM	SC.CH	SC.BA	NC.BE	VA.WA	MA.BA	MA.WE	MA.DU
FL.ST	-	0.002	0.007	0.002	0.002	0.001	0.001	0.001	0.001	0.001	0.007
FL.FP	0.721	-	NA	0.001	0.001	0.001	0.001	0.001	0.001	0.003	0.009
FL.CL	0.669	NA	-	0.004	0.005	0.001	0.001	0.001	0.001	0.034	0.030
FL.AM	0.783	1.000	1.000	-	1.000	0.341	0.356	0.284	0.001	0.028	0.085
SC.CH	0.806	1.000	1.000	−0.208	-	0.691	0.382	0.895	0.001	0.024	0.062
SC.BA	0.825	0.899	0.890	−0.095	−0.068	-	0.104	0.880	0.001	0.001	0.017
NC.BE	0.704	0.783	0.766	−0.043	−0.013	0.023	-	0.200	0.001	0.001	0.021
VA.WA	0.810	0.891	0.881	−0.094	−0.067	−0.033	0.019	-	0.001	0.003	0.023
MA.BA	0.879	1.000	1.000	1.000	1.000	0.922	0.822	0.914	-	0.233	0.169
MA.WE	0.667	0.867	0.809	0.709	0.761	0.766	0.600	0.744	0.336	-	1.000
MA.DU	0.635	0.858	0.784	0.638	0.708	0.714	0.518	0.687	0.512	−0.388	-

**Table 5:** Pairwise PhiST (below diagonal) and *p*-values (above diagonal) among regions.

	FL-Gulf	FL-Atlantic	FL-SC-NC	MA
FL-Gulf	-	0.001	0.001	0.001
FL-Atlantic	0.767	-	0.001	0.001
FL-SC-NC-VA	0.812	0.855	-	0.001
MA	0.794	0.911	0.825	-

## Data Availability

Code and datasets can be found at https://github.com/esotka/DiopatraCOI.git. COI sequences were uploaded to GenBank.
